# Measuring Faecal Glucocorticoid Metabolites to Assess Adrenocortical Activity in Reindeer

**DOI:** 10.3390/ani9110987

**Published:** 2019-11-18

**Authors:** Şeyda Özkan Gülzari, Grete Helen Meisfjord Jørgensen, Svein Morten Eilertsen, Inger Hansen, Snorre Bekkevold Hagen, Ida Fløystad, Rupert Palme

**Affiliations:** 1 Norwegian Institute of Bioeconomy Research, P.O. Box 115, 1431 Ås, Norway; seyda.ozkan@wur.nl (Ş.Ö.G.); svein.eilertsen@nibio.no (S.M.E.); inger.hansen@nibio.no (I.H.); snorre.hagen@nibio.no (S.B.H.); ida.floystad@nibio.no (I.F.); 2 Department of Biomedical Sciences, University of Veterinary Medicine, Veterinärplatz 1, A-1210 Vienna, Austria; rupert.palme@vetmeduni.ac.at

**Keywords:** reindeer, stress, glucocorticoids, validation

## Abstract

**Simple Summary:**

We validated an 11-oxoaetiocholanolone enzyme immunoassay for measuring faecal cortisol metabolites (FCMs) in reindeer. Samples were collected from eight male reindeer following adrenocorticotrophic hormone (ACTH) stimulation and from another group of reindeer during handling and calf marking. The overall FCM levels peaked after seven to eight hours in both locations, proving that the assay is suited to evaluate the adrenocortical activity in reindeer.

**Abstract:**

Several non-invasive methods for assessing stress responses have been developed and validated for many animal species. Due to species-specific differences in metabolism and excretion of stress hormones, methods should be validated for each species. The aim of this study was to conduct a physiological validation of an 11-oxoaetiocholanolone enzyme immunoassay (EIA) for measuring faecal cortisol metabolites (FCMs) in male reindeer by administration of adrenocorticotrophic hormone (ACTH; intramuscular, 0.25 mg per animal). A total of 317 samples were collected from eight male reindeer over a 44 h period at Tverrvatnet in Norway in mid-winter. In addition, 114 samples were collected from a group of reindeer during normal handling and calf marking at Stjernevatn in Norway. Following ACTH injection, FCM levels (median and range) were 568 (268–2415) ng/g after two hours, 2718 (414–8550) ng/g after seven hours and 918 (500–6931) ng/g after 24 h. Levels were significantly higher from seven hours onwards compared to earlier hours (*p* < 0.001). The FCM levels at Stjernevatn were significantly (*p* < 0.001) different before (samples collected zero to two hours; median: 479 ng/g) and after calf marking (eight to ten hours; median: 1469 ng/g). Identification of the faecal samples belonging to individual animals was conducted using DNA analysis across time. This study reports a successful validation of a non-invasive technique for measuring stress in reindeer, which can be applied in future studies in the fields of biology, ethology, ecology, animal conservation and welfare.

## 1. Introduction

The source of stress experienced by animals can be grouped into three categories: (i) physical due to disease or injury; (ii) physiological, for example, due to hunger or temperature control; and (iii) behavioural or psychological, for example, due to a change in living environment. Regardless of the cause, mammals exposed to stress are equipped with defense mechanisms, whereby catecholamines and glucocorticoids (GCs) are released from the adrenals [1]. In spite of their main task to eliminate the effects of the stressor, GCs, when secreted at high amounts over a prolonged time, may pose a risk to animal welfare. Therefore, measurement of cortisol as an indicator of stress is a useful approach in determining changes in health and biophysical parameters, even before the symptoms occur [2,3].

However, cortisol, like many other hormones, is secreted in an episodic and diurnal pattern in many animal species, making it difficult to draw conclusions from single samples. Further, handling animals during the collection of blood samples exposes them to additional stress and may produce results that may reflect the consequences of handling procedures. Therefore, non-invasive and “feedback-free” sampling methods, such as collection of faeces, are preferred [2,4]. Concentrations of faecal cortisol metabolites (FCMs) reflect secreted GCs better than plasma cortisol concentrations estimated at the time of blood collection, providing a reliable approach for measuring adrenocortical function [5,6]. 

Reindeer (*Rangifer Tarandus Tarandus L.*) are free-ranging, semi-domesticated animals that are not accustomed to being handled by humans in the same way as cattle, sheep or pigs [7]. Gathering, sorting and handling may, therefore, result in increased levels of blood cortisol and urea concentrations [8]. Stress results in increased pH levels and reduced glycogen reserves in the meat, compromising the meat quality [9] and, hence, the shelf life of reindeer products [10,11].

A physiological validation of a non-invasive method aims at specifically stimulating the adrenal glands to release GCs into circulation, which should be well captured in the excreted faecal metabolites [12]. Such validations also yield information about the species-specific time delay between ACTH stimuli and detection of increased FCM levels—a time delay that also corresponds roughly to gut passage time [5]. Species differences in metabolism and excretion of glucocorticoids require that each method is validated for each species and sex [12]. The determination of faecal stress hormone metabolites in mammals and birds has been performed in many species [5,13]. However, it appears that only Ashley et al. [14] have previously performed an ACTH test in reindeer. Since their study did not show an expressed response in FCMs even after a high dose of ACTH, a full validation of such a method in reindeer still appears to be lacking. Thus, this paper aims to evaluate the physiological relevance of an enzyme immunoassay (EIA) for determining FCMs in reindeer through an ACTH challenge, in addition to assessing FCM concentrations of reindeer during calf marking as a biological validation. 

## 2. Materials and Methods

In order to obtain enough reference data to validate the FCMs, two separate studies were performed. The ACTH challenge test was done on eight animals in a controlled fence facility at Tverrvatnet and collection of faecal samples for biological validation was performed for a whole flock of reindeer, representing different ages and both sexes, at Stjernevatn. 

### 2.1. Reindeer (Both Regions) and ACTH Challenge Test (Only Tverrvatnet)

At Tverrvatnet, eight reindeer males (≥1.5 years of age) were injected intramuscularly with 1 mL adrenocorticotrophic hormone (ACTH; 0.25 mg/mL: Synacthen^®^; CD Pharma Srl & CD Pharmaceuticals AB, Sweden) in the neck area in front of the shoulder. After the injection of ACTH, animals were moved to a designated fenced area where they were not disturbed except for the collection of samples or refilling of food. At Stjernevatn, on the other hand, the animals were gathered for calf marking. The herd consisted of females (lactating and non-lactating), males, yearlings and calves.

### 2.2. Study Area and Field Conditions

The physiological validation study was carried out at Tverrvatnet (66° N) in Rana municipality in Nordland County in Norway in January 2018. The area was covered with snow and the ambient temperature ranged from −10 to −15 °C. In addition, a biological validation was performed at Stjernevatn (70° N) in Tana municipality in Finnmark County in Norway in August 2017. The ambient temperature at Stjernevatn ranged from 5 to 13 °C.

### 2.3. Faecal Pellet Collection

In both locations, faecal samples were collected opportunistically, resulting in a challenge to quickly discriminate the samples belonging to adults or calves. Flasko et al. [15] suggest that pellet size could be used as a significant criterion to distinguish calves from adults in wild Canadian caribou (*Rangifer tarandus caribou*). Therefore, to overcome the problem, we allocated the samples with an apparent small pellet size to calves. 

#### 2.3.1. Tverrvatnet

To avoid confounding effects of separation stress, the experimental animals at Tverrvatnet were not moved to the experimental enclosure before the injection of ACTH. Faecal samples were collected from the group instead of from each individual reindeer to avoid disturbing the animals and creating additional stress. The identification of individual animals was difficult due to the limited daylight in this area in winter. Since the DNA analysis conducted later revealed the individual identity of the anonymous samples, the above-mentioned challenges were not seen as an obstacle.

The collection of samples started two hours after the injection of ACTH and was repeated every hour until the 12th h and every second hour until 30 h and finished by taking a final sample at 44 h. All samples (n = 317) were collected in plastic bags or in disposable gloves and frozen at ambient temperature (between −10 and −20 °C). Samples were kept frozen at −18 °C until extraction in the laboratory. 

#### 2.3.2. Stjernevatn

The sample (n = 114) collection took place from 10:30 to 20:35, forming a total of around 10 h, during two phases: (i) separation of males and non-lactating females from lactating females and calves; and (ii) earmarking of calves. In the first phase, a subgroup of 12 to 30 reindeer were moved into the handling fence where animals were caught by hand and examined by the reindeer herders. The females, which were lactating, were marked with a colour spray and later released back into the subgroup where they eventually remained together with all calves. The males or non-lactating females were moved to adjacent holding pens for separation. Each batch took around 2–8 min to process, depending on the batch size. This phase took approximately five hours, during which a total of 800–900 animals were processed. Faecal samples collected during the first two hours of the separation process were considered as baseline (n = 51, of which n = 31 from known calves). 

The second phase of faeces collection was done when calves and lactating females were brought back to the fence for earmarking. In the same way as before, smaller subgroups were taken into the work fence in batches and processed there. Between batches, researchers collected fresh faecal samples from the ground (n = 63, of which n = 34 from known calves) to determine potential changes in FCM concentrations as an effect of handling. This phase lasted 2.5 h. At Stjernevatn, faecal samples could not be attributed to individual reindeer, but stools with smaller pellets were marked as belonging to calves, as described above.

### 2.4. Analysis of FCMs

Some of the samples from Tverrvatnet were already covered in snow and, to avoid the extra weight snow adds, all samples were oven-dried at 75 °C for 24 h. The dried samples were ground and homogenized using a mortar and pestle, and 0.2 g of each sample was weighed. Steroid extraction was performed according to Palme et al. [16] and Palme [17]. For suspension of samples, 4 mL 99.9% methanol was mixed with 1 mL distilled water and added to the 0.2 g faecal sample. The mixture was hand-vortexed for 1–2 min followed by centrifugation at 2.500 g for 15 min. A 0.5 mL aliquot of each supernatant was transferred to microtubes and stored at −18 °C until analysis. 

FCMs were measured in aliquots (after further 1:10 dilution with assay buffer) of the extracts, utilizing a group-specific enzyme immunoassay (11-oxoaetiocholanolone EIA), previously described in detail by Möstl et al. [18], which was found to be well suited in various ruminant species [5]. Intra- and inter-coefficients of variation were below 10 and 15%, respectively.

### 2.5. DNA Analysis

To identify the individual in the ACTH challenge test that the faecal samples belonged to, we performed DNA analysis on 303 of the 317 samples from Tverrvatnet. A total of 14 samples were discarded at this point, due to low quality.

#### 2.5.1. DNA Extraction

One faecal pellet from each of the 303 faecal samples was transferred to a stool collection tube containing 8 mL stool DNA stabilizer. The DNA was then extracted using PSP Spin Stool DNA Plus Kit (Stratec, Birkenfeld, Germany) following the manufacturers protocol.

#### 2.5.2. PCR Amplification

To reliably assign samples to individual reindeers, we used eight dinucleotide microsatellite markers that were sorted into two multiplex assays for efficient genotyping (multiplex 1 and 2); six of the markers were taken from Wilson et al. [19] (RT1, RT6, RT13, RT20, RT27, RT30) and two from Røed and Midthjell [20] (NVHRT22, NVHRT46). The markers can be found in GeneBank using the accession numbers found in Table 1. The forward-primers were labeled with one of three fluorescent dyes (6FAM, NED or PET, Table 1). In addition, the ′pigtale′ sequence [21] was added to the 5′ end of one of the reverse primers to facilitate accurate genotyping.

The PCR reactions were carried out in 10 µL reaction volume: 5 µL 2× multiplex PCR master mix (Qiagen Multiplex kit), 0.05 µg/µL BSA (NEB) and adjusted primer set concentrations (Table 1). The PCR conditions for both multiplexes were 10 min at 95 °C, 35 cycles of 30 s at 94 °C, 30 s at 58 °C, 1 min 72 °C and a final extension for 45 min at 72 °C.

The PCR products (1 µL) were then mixed with Genescan 500 LIZ (Applied Biosystems) size standard (0.24 µL) and Hi-Di formamide (10.00 µL), following a 2 min denaturation at 95 °C on a 2720 Thermal cycler. Capillary electrophoresis was carried out on an ABI 3730 DNA Analyzer (Applied Biosystems). The POP-7™ Polymer was used as a separation matrix and the sample injection times were set to 4 s/2 kv. The PCR fragments were analyzed in GeneMapper 4.1 (Applied Biosystems). The alleles were automatically scored and then manually checked. 

To check for possible contamination, every eighth sample added was a negative control. Negative controls contained all of the PCR master-mix components except the DNA template (water was added instead of DNA). Four samples (two tissue- and two scat samples) from previously known reindeer were added as positive controls. Two samples were excluded from the analysis due to an error in registration, leaving 303 samples for the genetic analysis. The combination of the eight markers comprised the genetic profile of an individual reindeer. The samples with the same genetic profile were grouped together and linked to the same individual reindeer.

### 2.6. Animal Welfare

This study was conducted in accordance with the regulation for use of animals in experiments, adopted by the Norwegian Ministry of Agriculture and Food, and approved by the Ethics Commission on Animal Use by the Norwegian Food and Safety Authority, application number (FOTS ID) 12274 on 03.04.2017. It complies with the EU Directive 2010/63/EU on the use of experimental animals, which was incorporated to the EEA Agreement in May 2015.

### 2.7. Statistical Analysis

Means, medians and standard deviations (SD) were calculated using data analysis functions in Microsoft Office^®^ Excel. Data were investigated for normal distribution and *log* transformed before analysis. The analysis was generated using SAS software, Version 9.4 of the SAS system for Windows version 6.2.92002 [22].

#### 2.7.1. Samples from Tverrvatnet

First, a non-parametric comparison was performed using the npar1way command with “Animal” as a class variable and “FCM” as the response variable. Using transformed data, the effect of “hour since ACTH administration” on FCM concentrations was analyzed using a mixed model of analysis of variance with “Hour” (1–44) and “Animal” (1–8) as class variables. “Animal” was specified as a random effect and degrees of freedom were calculated using the Satterthwaite’s approximation. Differences between means were investigated using the LSmeans command with the Tukey–Kramer approximation.

#### 2.7.2. Samples from Stjernevatn

Using transformed data, the effect of handling on FCM levels was investigated using a general linear model of analysis of variance with “Time of day” (morning or afternoon, reflecting the time periods 0–2 h or 8–10 h after handling) and “Age” (adult/calf) as class variables.

## 3. Results

### 3.1. Controlled Experiment with ACTH Challenge on Eight Reindeer (Tverrvatnet)

#### 3.1.1. Identification of Individual Animals

**Figure 1:**
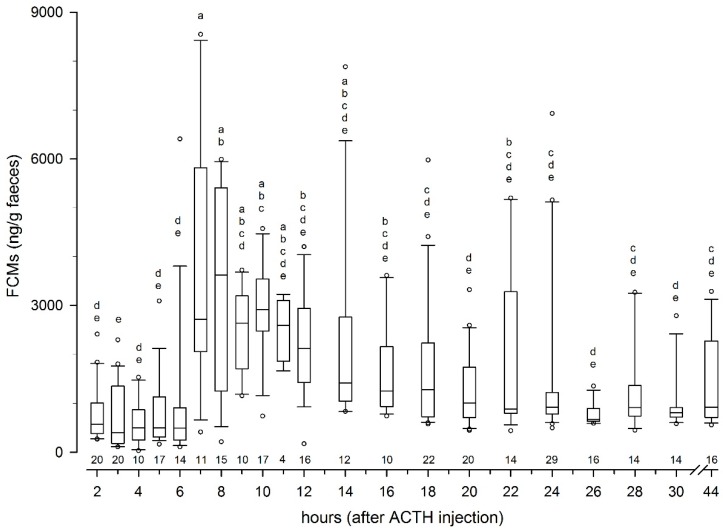
Box-plot graphs of levels of faecal cortisol metabolites of all samples as a function of hours after ACTH administration. Hours with dissimilar letters had significantly different (*p* < 0.05) concentrations. Above the x-axis, the respective numbers of samples are given.

Of the 303 faecal samples analyzed, 48 (16%) were negative for all eight microsatelite markers and 255 (84%) were positive for at least one STR-marker. Among the 255 samples, eight unique genetic profiles were found, representing the eight individual reindeer. A total of 28 of the positive samples (11%) could not be assigned to one of the eight unique profiles, due to the lesser quality of the samples and/or the lack of private alleles or unique allele combinations. The 227 samples (89%) were given an identity based on the eight unique genetic profiles that make up the eight reindeer individuals (Figure 1). As a result, 54 of the samples were assigned to individual 1; 32 samples to individual 2; 15 samples to individual 3; 39 samples to individual 4; 17 samples to individual 5; 16 samples to individual 6; 34 samples to individual 7; and 20 samples to individual 8.

#### 3.1.2. Results from the ACTH Challenge Test

Median (range) FCM levels from two to six hours after injection of ACTH were 505 (34–6408 ng/g). Overall, FCM concentrations peaked at 7 h and slowly decreased afterwards (including high values in some intervals; Figure 1).

**Figure 2:**
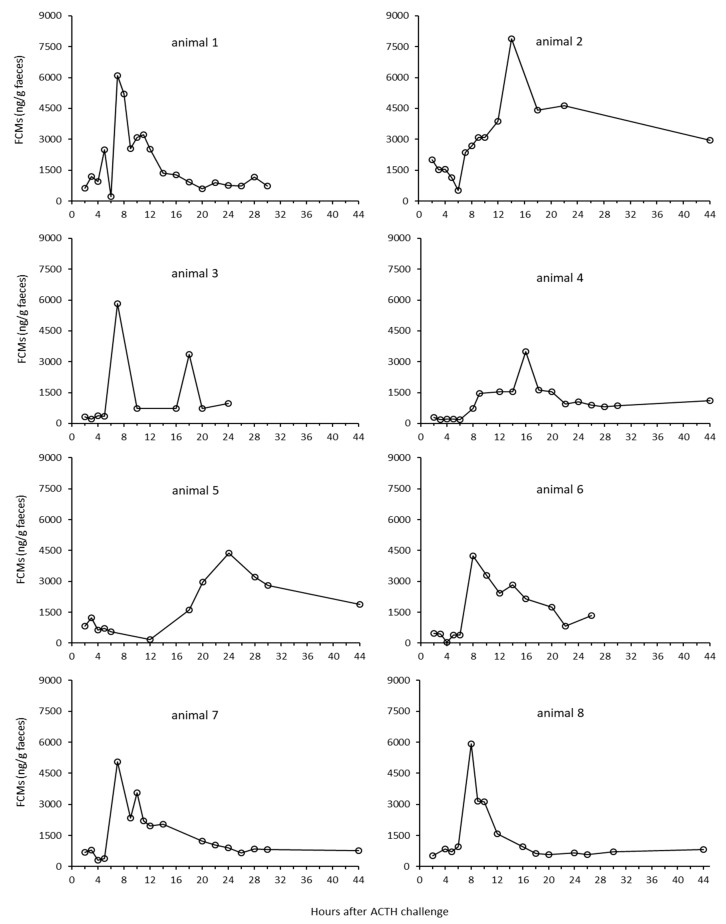
Concentration of faecal cortisol metabolites (FCMs) from identified samples of the eight individual animals at Tverrvatnet, shown as a function of hours after ACTH challenge.

When expressed at individual animal levels, the FCM concentrations align with the herd level for five animals where peak levels ranged from 5000 ng to 8000 ng/g faeces. As can be seen in Figure 2, animal numbers 2, 4 and 5 reached their peak values later than the other individuals. There were three unidentified high concentration (6408, 5409 and 3721 ng/g faeces) samples (6–9 h), which could have been those animals’ (e.g., animal 5) early peak samples. Individual peak samples were about 6.3 (2.0–16.8) times higher (median 3359; range 2352–5361 ng/g) than respective baselines (0–6 h; 685; 315–1079 ng/g faeces).

### 3.2. Results from the Biological Validation (Normal Handling and Calf Marking at Stjernevatn)

**Figure 3:**
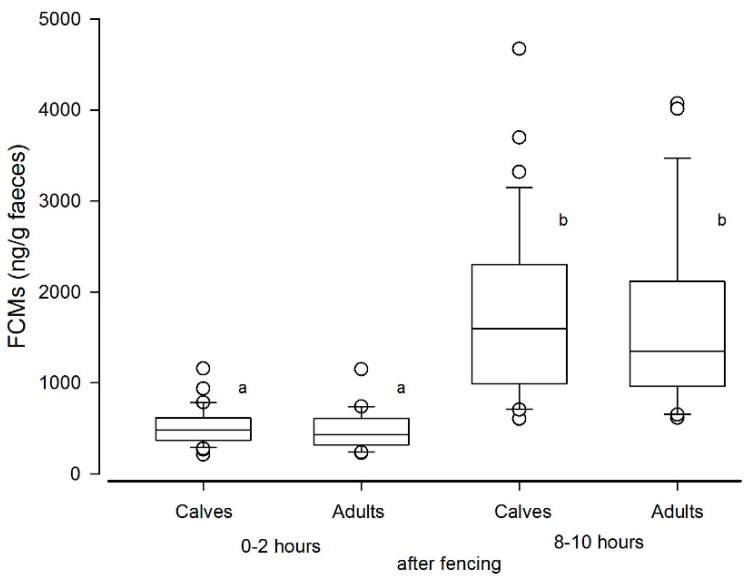
Concentrations of faecal cortisol metabolites (FCMs) in reindeer collected immediately after fencing (0–2 h; morning, n = 51 where 31 were calves and 20 adults) and later after handling and calf marking (8–10 h; evening, n = 63 where 34 were calves and 29 adults). Dissimilar letters indicate significant (*p* < 0.001) differences.

The highest values of cortisol metabolites were found around eight hours after the human activity started within the fences. FCM concentrations (median; range) in reindeer (Figure 3) were significantly different between morning (480; 212–1159 ng/g faeces) and evening (1469; 605–4673 ng/g faeces) sampling (F = 116.9; *p* < 0.001). Calves and adults did not show any significant differences in FCM levels (F = 1.35; *p* = 0.25). A total of 65 samples were from calves and 49 samples were from adult reindeer. In the first phase (0–2 h) adult samples were from males and females, non-lactating and lactating. In the second phase (8–10 h) samples from adult reindeer came only from lactating females with calves at foot.

## 4. Discussion

### 4.1. Non-Invasive Versus Non-Disturbing

Even though faeces collection is a non-invasive approach compared to blood collection, it may not be non-disturbing, because sample collection can trigger a stress response in animals that are not used to close human presence [4]. This is especially true with herd animals such as reindeer. Since stress is conducive to changes in welfare, production, physiology, health and mortality [23], even slow but repetitive entries into the fence, prompting sudden movements or alertness of the animals, may increase cortisol levels. This will not affect FCM levels in the sample collected immediately but may very well result in higher levels in serial samples collected later on [5]. Thus, the experimental animals receiving ACTH in Tverrvatnet may have already had higher cortisol levels, due to the earlier handling by the reindeer herder moving the animals in the fence. The delayed decrease in FCM levels may be attributed to such a disturbance, because, during the first phase, samples were taken from the herd every hour. 

Even though the sample collection at Stjernevatn started once the animals were moved to the working fence, the gathering earlier in the morning may also have influenced or even exacerbated the stress levels of the animals. However, since the time lag between the time the work started in the working fence and the gathering was less than seven hours, the 0–2 h samples most likely reflect true FCM baseline levels. 

Sampling protocols, based on earlier experience in similar studies, were prepared in detail before the data collection started. A total number of 317 samples were collected over 44 h in the ACTH challenge test, which should capture any peak levels in FCMs. Earlier studies have demonstrated a time lag of FCM in reindeer of 8 to 24 h [14]. In the biological validation, a total number of 114 samples were collected over 10 h, which could be too short to detect late peak levels. After handling was completed, it was usual practice to release the animals back to pasture. Holding them much longer in fence would, therefore, not be justified in the private herd visited.

### 4.2. Peak FCM Levels

Following the ACTH challenge, all animals showed well expressed peak FCM levels which were about 630% above their respective baseline levels. This is the first time such a non-invasive method to measure FCM levels in reindeer has been successfully validated. Comparing absolute values in the present study with earlier studies not using the same immunoassays is not relevant, as the different antibodies pick up different cortisol metabolites and to a different degree [5]. The FCM levels peaked seven to eight hours after stimulating the adrenals. This was quicker than reported delay times for deer or goats [24,25], but closer to those found in other ruminants (10–12 h), such as sheep or cattle [6]. However, our results correspond well with findings in caribou, where a significant (but not comparably expressed) increase in FCM was found eight hours after an ACTH challenge [14]. 

In the biological validation experiment performed at Stjernevatn, the FCM levels eight to ten hours after handling showed more than a three-fold rise compared to baseline levels (samples collected after 0–2 h). This indicates that our method is sensitive enough to detect the effect of handling stress. However, the peak FCM levels were lower when compared to the ACTH challenge test. A study by Carlsson et al. [26] found no increase in FCM levels eight hours after handling stress, but their method was not validated for this species. This underlines the importance of performing validation studies in every animal species and validating each method of FCM analysis against baseline values found under practical and representative conditions.

### 4.3. Effects of Sex and Individual

At Tverrvatnet, only males were used, because the females were pregnant. Ashley et al. [14] found that female reindeer showed a prolonged response to a very high ACTH dose (8 IU/kg; possibly about 15–16 times higher than the one used here (about 25 IU/animal), assuming the animals weighed about 45–50 kg, whereas males did not have this response. Faecal samples were collected from both males and females in the first collection period at Stjernevatn. In the second period, only lactating females and calves were present. Sex and age may have an influence on FCM levels [5,12]. However, the variation in our dataset from the biological validation was small, as shown in Figure 3. Furthermore, the statistical analysis showed no difference in FCMs between calves and adults, thus making such an influence less likely. 

There were quite large individual differences in the FCM response for the ACTH challenge test at Tverrvatnet. Some animals (animals 1, 3, 6, 7, 8) had an early peak (about seven hours after ACTH injection), in others (animals 2, 4 and 5) it occurred later (after 12, 14 and up 24 h, respectively). The reasons for this might be manifold. Differences in the resorption of the ACTH could have played a role. If the needle hits the muscle close to a blood vessel, the response should be quicker (immediate) than if the ACTH injection was further away from a vessel. In case of the latter, the increase could be delayed. Another explanation might be that earlier peaks were missed in those animals. This is likely true in animals (e.g., animal 5) where samples were probably missed, especially as high concentration samples of unknown identity were present in that time window (7–8 h). In addition, the enterohepatic recirculation, which has been proven in ruminants [27], could have caused secondary peaks, especially when they were smaller (e.g., animals 1 and 3). However, median delay times in FCM peaks found in the biological validation at Stjernevatn were also seven hours, which suggests that shorter delay times in faecal peak excretion are more likely found in reindeer.

### 4.4. Identification of Unknown Samples

Being able to document the individual responses by using DNA analysis added extra value to the results because these individual patterns would have been hidden in anonymous sampling. Non-invasive genetic sampling methods have become widely used in genetic studies and have been shown to be a reliable approach for genetic studies in reindeer [28,29,30]. It has also been shown that combining genetic analysis with FCM measurements is a useful approach to document individuals and their sex, and that ignoring this information could lead to erroneous conclusions when addressing stress levels in animal populations [31,32].

In this study, we have used non-invasive genetic sampling without any previous knowledge of the genotypes of the eight individuals involved in the experiment and achieved a high success rate in assigning samples to genotypes/individuals across the duration of the study. This shows that the technique can be a useful addition to other scientific fields, to ensure reliable tracking of individuals across time and reduced degree of disturbance (i.e., less human-induced stress responses in the animals). Since we already knew the sex of the eight reindeer individuals, a molecular sexing was not performed, but is advised in future studies without this knowledge, whenever sex can be expected to be an important explanatory factor.

Samples collected by non-invasive methods, such as faecal samples, are less likely to give complete profiles due to low quality and/or quantity (i.e., allelic dropout or fragmented DNA) compared to blood and tissue samples [33,34,35]. When possible, we therefore recommend including control samples (tissue or blood) from all individuals, collected either before or after the experiment, to confirm their genetic profiles. This would facilitate unambiguous assignment of samples to genetic profiles/individuals and thus contribute to a higher percentage of the samples being available for the primary analysis, e.g., FCM or other applications. 

### 4.5. Ways Forward

Using an animal as its own control in the statistical analysis gave us the opportunity to use fewer animals in the ACTH challenge test. This is in line with the three R’s from the ethical code of conduct for animal experiments. 

Reindeer are free-ranging ungulates that move over large distances during the production year. Factors including natural and climatic changes affect their welfare. FCM may be used as an objective measure of stress load under different handling procedures, or when human activity interferes with grazing areas and reindeer habitats. The non-invasive faecal sample collection from the ground is of great benefit, when working with this fearful and flock-dependent species. Another benefit is the fact that we have discovered a time-lag of around seven to eight hours between the moment a stressor is experienced until peak FCM levels are reached. Knowing this is imperative when investigating the effects of known potential stressors and comparing them to FCM levels collected before, during and after periods of interference.

## 5. Conclusions

We have successfully validated an 11-oxoaetiocholanolone EIA for measuring FCMs in order to evaluate adrenocortical activity in reindeer. We found a time-lag of approximately seven to eight hours between the ACTH challenge and peak FCM levels. A similar time-lag was observed when samples were taken after human handling and calf marking in the fence. The method proved biologically sensitive and could be of great value as a tool for welfare assessment of different treatments and handling procedures, and also for evaluating the negative effects of human disturbance in natural pasture areas. As collecting individual faecal samples in a group of housed, semi-free species like reindeer may disturb the animals, combining FCM analysis with genetic data proved useful to overcome the problem of anonymous sampling. 

## Figures and Tables

**Table 1:** Primer characteristics and PCR conditions for the eight microsatellite markers in *Rangifer tarandus.*

Multiplex Assays	Microsatellite Marker	Primer Sequences (5′–3′)	Repeat Motif	PCR Concentration and Fluorescent Dye	Source	GeneBank Accession No.
1	NVHRT22	F: GTATTCTTGCCAGGAAAAACC	CA	0.1 µM, NED	Røed and Midthjell [20]	AF068208
		R: GTTGCTTCAGTGCTCTCAGAT				
	RT1	F: TGCCTTCTTTCATCCAACAA	GT	0.2 µM, FAM	Wilson et al. [19]	U90737
		R: CATCTTCCCATCCTCTTTAC				
	RT20	F: GCAGAAGAGTGAGTGTGAGT	GT	0.2 µM, NED	Wilson et al. [19]	U90744
		R: GTTTCTTGTTGTATTTTGGACCTTT				
	RT13	F: GCCCAGTGTTAGGAAAGAAG	GT	0.2 µM, FAM	Wilson et al. [19]	U90743
		R: CATCCCAGAACAGGAGTGAG				
2	NVHRT46	F: CCGACTGAAGTGACCAAG	CA	0.2 µM, FAM	Røed and Midthjell [20]	AF068213
		R: TGTTGAGAGGATTGATAAG				
	RT6	F: TTCCTCTTACTCATTCTTGG	GT	0.4 µM, NED	Wilson et al. [19]	U90739
		R: CGGATTTTGAGACTGTTAC				
	RT30	F: CACTTGGCTTTTGGACTTA	GT	0.3 µM, NED	Wilson et al. [19]	U90749
		R: CTGGTGTATGTATGCACACT				
	RT27	F: CCAAAGACCCAACAGATG	GT	0.2 µM, PET	Wilson et al. [19]	U90748
		R: TTGTAACACAGCAAAAGCATT				

”Pigtail” sequence added to the 5′ end of the primer according to Brownstein et al. [21].
